# Fatal Pediatric COVID-19 Case With Seizures and Fulminant Cerebral Edema

**DOI:** 10.1177/2329048X211022532

**Published:** 2021-06-14

**Authors:** Siddharth Ninan, Peyton Thompson, Timothy Gershon, Natalie Ford, William Mills, Valerie Jewells, Leigh Thorne, Katherine Saunders, Thomas Bouldin, Jason R. Smedberg, Melissa B. Miller, Eveline Wu, Alyssa Tilly, Jeremy Sites, Daniel Lercher, Katherine Clement, Tracie Walker, Paul Shea, Benny Joyner, Rebecca Smith

**Affiliations:** 1 Department of Neurology, University of North Carolina at Chapel Hill, NC, USA; 2 Department of Pediatrics, Division of Infectious Diseases, University of North Carolina at Chapel Hill, NC, USA; 3 Department of Pediatrics, University of North Carolina at Chapel Hill, NC, USA; 4 Department of Pediatrics, Division of Emergency Medicine, University of North Carolina at Chapel Hill, NC, USA; 5 Department of Radiology, University of North Carolina at Chapel Hill, NC, USA; 6 Department of Pathology and Laboratory Medicine, University of North Carolina at Chapel Hill, NC, USA; 7 Clinical Microbiology Laboratory, McLendon Clinical Laboratories, UNC Medical Center, Chapel Hill, NC; 8 Department of Pediatrics, Division of Allergy, Immunology and Rheumatology, University of North Carolina at Chapel Hill, NC, USA; 9 Departments of Medicine and Pediatrics, Palliative Care Program, University of North Carolina at Chapel Hill, NC, USA; 10 Department of Pediatrics, Division of Critical Care, University of North Carolina at Chapel Hill, NC, USA

**Keywords:** COVID-19, pediatric, seizures, cerebral edema

## Abstract

The novel coronavirus, SARS-CoV-2, can present with a wide range of neurological manifestations, in both adult and pediatric populations. We describe here the case of a previously healthy 8-year-old girl who presented with seizures, encephalopathy, and rapidly progressive, diffuse, and ultimately fatal cerebral edema in the setting of acute COVID-19 infection. CSF analysis, microbiological testing, and neuropathology yielded no evidence of infection or acute inflammation within the central nervous system. Acute fulminant cerebral edema (AFCE) is an often fatal pediatric clinical entity consisting of fever, encephalopathy, and new-onset seizures followed by rapid, diffuse, and medically-refractory cerebral edema. AFCE occurs as a rare complication of a variety of common pediatric infections and a CNS pathogen is identified in only a minority of cases, suggesting a para-infectious mechanism of edema. This report suggests that COVID-19 infection can precipitate AFCE, and highlights the need for high suspicion and early recognition thereof.

COVID-19, caused by the novel SARS-CoV-2 virus, emerged in December 2019 causing widespread morbidity and mortality. Coronaviruses have associated neurological manifestations, as noted during the SARS (2002) and MERS (2012) epidemics.^1^ Multiple neurological manifestations of COVID-19 have been described in children, including encephalitis/encephalopathy, seizure-like activity, and peripheral weakness.^2–7^

Acute fulminant cerebral edema (AFCE) is a poorly understood clinical entity characterized by fever, encephalopathy and often new seizures followed by rapid progression to diffuse cerebral edema, with potentially fatal outcomes.^8,9^ Postmortem examination of the brain in fatal cases of AFCE confirms diffuse brain edema but does not reveal an underlying cause, such as meningitis, encephalitis, brain infarcts, hypoxic-ischemic encephalopathy, or toxic/metabolic encephalopathy.^8^ We report a child with acute seizures and acute fulminant cerebral edema in the context of COVID-19 infection.

## Case History

 A previously healthy 8-year-old Latina female presented to our emergency department (ED) with 1 day of fever (T_max_ 100.4ºF), lethargy, myalgias, anorexia, and new-onset seizure-like activity. She was diagnosed with group A streptococcal (GAS) pharyngitis and COVID-19 at an urgent care clinic, just prior to presenting to the ED. En route to retrieve antibiotics for GAS, she developed emesis, altered consciousness, and had a 2-minute, self-resolving period of seizure-like activity, described as generalized convulsions and drooling. The patient was previously healthy without prior hospitalizations, up-to-date on immunizations, and without known head trauma, drug allergies, inadvertent ingestions, or daily medications.

Her vital signs in the ED were: rectal temperature 38.9°C, heart rate 88, respiratory rate 33, blood pressure 92/69, SpO2 91% on room air. She was awake but drowsy and in no acute distress. Neurological examination revealed mild confusion, mildly reduced verbal output with appropriate responses to simple questions, inconsistent ability to follow commands, no focal motor deficits and no meningeal signs. Skin examination revealed a pink, fine macular rash wrapping around her trunk and upper extremities, sparing her palms/soles. She had posterior pharyngeal erythema without tonsillar hypertrophy or exudate. The remainder of the examination was unremarkable.

The patient remained stable for a 2-hour period until resumption of seizure activity, consisting of 1 minute of generalized convulsions followed by decorticate posturing of the limbs, trismus, right head version, and alternating right and left eye deviation. Pupils remained equal, round and reactive to light throughout. She had an episode of emesis during the seizure with suspected aspiration; her SpO2 dropped as low as 86% but quickly corrected to > 92% on 4 L O2. Her seizures were unresponsive to 2 doses of intravenous lorazepam (0.1 mg/kg), but terminated with a loading bolus of levetiracetam (60 mg/kg) after 25 minutes.

Initial laboratory evaluation revealed a normocytic anemia, slightly elevated AST, and slightly elevated C-reactive protein (Table 1). A nasopharyngeal swab for SARS-CoV-2 RT-PCR returned positive. After a blood culture was collected (which ultimately revealed no growth), broad-spectrum antimicrobials (vancomycin, ceftriaxone, and acyclovir) were initiated for possible meningitis. Chest X-ray showed prominent right perihilar and bibasilar lung markings. A non-contrast head CT **following her seizure episode in the ED** showed sulcal loss suggesting global edema, without evidence of herniation. Given her failure to return to her baseline mental status she was admitted to the Pediatric Intensive Care Unit (PICU) for continuous video EEG (cvEEG) and closer monitoring.

**Table 1.** Initial Laboratory^*^ and Microbiological Findings.

Parameter	Patient’s value	Units	Reference value
Complete Blood Count			
White blood cell count	6.9	10^9^/L	4.5-13.0
Hemoglobin	*10.6*	g/dL	13.0-16.0
MCV	83.4	fL	78.0-98.0
Platelet count	183	10^9^/L	150-440
Complete metabolic panel			
Sodium	143	mmol/L	135-145
Potassium	4.3	mmol/L	3.4-4.7
Chloride	105	mmol/L	98-107
CO2	22.0	mmol/L	22.0-30.0
BUN	12	mg/dL	5-17
Creatinine	0.39	mg/dL	0.30-0.90
Anion gap	*16*	mmol/L	7-15
Glucose	128	mg/dL	70-179
Calcium	9.0	mg/dL	8.8-10.8
Magnesium	2.0	mg/dL	1.6-2.2
Phosphorus	*3.9*	mg/dL	4.0-5.7
Albumin	4.1	g/dL	3.5-5.0
Total Protein	7.9	g/dL	6.5-8.3
Total Bilirubin	0.2	mg/dL	0.0 -1.2
AST	*53*	U/L	10-40
ALT	37	U/L	<50
Alkaline Phosphatase	132	U/L	130-560
Cardiac Enzymes			
Troponin I	<0.034	ng/mL	<0.034
Pro-BNP	*547.0*	pg/mL	0.0-178.0
ESR	19.0	mm/h	0-20
CRP	*19.1*	mg/L	<10.0
LDH	742	U/L	380-770
Coagulation Panel			
PT	*14.4*	sec	10.2-13.1
INR	1.25	N/A	N/A
APTT	36.5	sec	25.9-39.5
D-Dimer	*3,493*	DDU	<230
Fibrinogen	316	mg/dL	177-386
Infectious Disease Test Results			
SARS-CoV-2 PCR (nasopharyngeal)	*Positive*	N/A	Negative
SARS-CoV-2 PCR (CSF)	Negative	N/A	Negative
Blood culture	No growth	N/A	No growth
CSF culture	No growth	N/A	No growth
4th generation HIV Ag/Ab	Negative	N/A	Negative
CMV PCR (serum and CSF)	Undetectable	N/A	Undetectable
EBV PCR (serum and CSF)	Undetectable	N/A	Undetectable
HHV-6 PCR (serum and CSF)	Undetectable	N/A	Undetectable
HSV 1/2 PCR (serum and CSF)	Undetectable	N/A	Undetectable
VZV PCR (serum and CSF)	Undetectable	N/A	Undetectable
Enterovirus PCR (serum and CSF)	Undetectable	N/A	Undetectable
Arbovirus panel (CSF)	Negative	N/A	Negative
Parvovirus B-19 PCR (serum)	Undetectable	N/A	Undetectable
Bartonella antibody panel (serum)	Negative	N/A	Negative
LCMV PCR/antibody (serum and CSF)	Negative	N/A	Negative
Naegleria PCR (CSF)	Undetectable	N/A	Undetectable
Balamuthia PCR (CSF)	Undetectable	N/A	Undetectable
Acanthamoeba PCR (CSF)	Undetectable	N/A	Undetectable
Baylisascaris procyonis Ab (serum)	Negative	N/A	Negative
Postmortem Microbiological Results			
Aerobic culture, left lung	*Candida parapsilosis* ^1^, *Lactobacillus*	N/A	No growth
Aerobic culture, right lung	No growth	N/A	No growth
Aerobic culture, brain tissue	<1+ *Candida parapsilosis* ^2^	N/A	No growth
Aerobic/anaerobic culture, spleen	No growth	N/A	No growth
AFB smear/culture, left lung	No growth	N/A	No growth
AFB smear/culture, right lung	No growth	N/A	No growth
AFB smear/culture, brain tissue	No growth	N/A	No growth
Fungal culture, left lung	4+ *Candida parapsilosis* ^1^	N/A	No growth
Fungal culture, right lung	No growth	N/A	No growth
Fungal culture, brain tissue	No growth	N/A	No growth
Respiratory pathogen panel, left lung	Negative	N/A	Negative
Respiratory pathogen panel, right lung	Negative	N/A	Negative
SARS-CoV-2 PCR (blood)	Negative	N/A	Negative
SARS-CoV-2 PCR (brain tissue)	Negative	N/A	Negative
SARS-CoV-2 PCR (left lung)	Negative	N/A	Negative
SARS-CoV-2 PCR (right lung)	Negative	N/A	Negative

^*^ Initial laboratory findings obtained while patient was in the emergency department.

^1^This fungal growth was likely related to the patient’s known aspiration event.

^2^Growth from aerobic culture of brain tissue was believed to be contamination from lung tissue at time of autopsy; growth in only 1 of 2 aerobic cultures from brain tissue.

**Figure 1.**
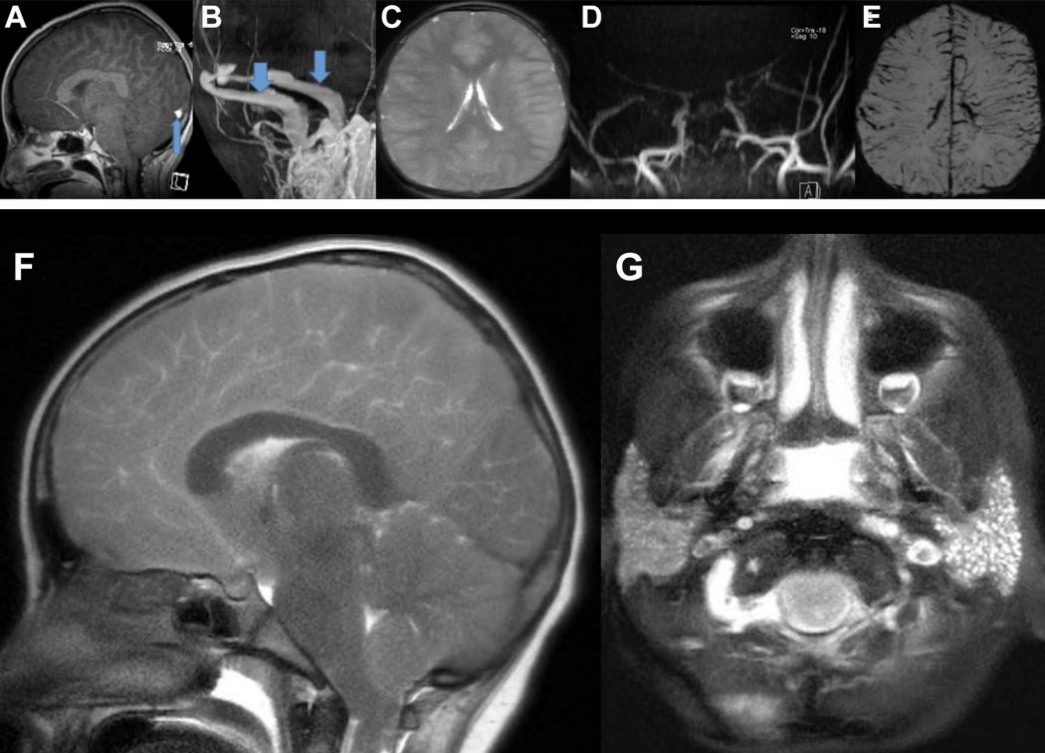
Brain imaging (MRI/MRA/MRV). day #1 brain MRI (A) sagittal T1 image demonstrates downward herniation with decreased size of the suprasellar cistern, posterior fossa crowding causing brainstem compression against the clivus and developing tonsillar herniation. (B) MRV demonstrates patent jugular veins and transverse sinuses without thrombosis. The sagittal sinus and deep venous system are not visible. (C) T2 axial images reveal diffuse cortical swelling mild, global gray matter hyperintensity, and slit-like ventricles suggesting brain edema. (D) coronal MRA image demonstrates near complete or complete loss ACA, MCA, PCA and basilar flow. (E) susceptibility-weighted imaging shows exaggerated cortical veins consistent with engorgement vs thrombosis. the subsequent MRI sagittal T1 (F), day #2 demonstrates tonsillar herniation down to the level of C2. MRIs on day #1 and 2 revealed bilateral parotid cysts (G), suggesting parotitis, which has been reported in cases of COVID-19 infection.^21,22^

On arrival to the PICU the patient was minimally responsive, with intermittent decerebrate posturing, rhythmic upper extremity flexion, and twitching of all 4 extremities; pupils remained equal, round, and reactive to light bilaterally. In response to her intermittent rhythmic movements, she received 3 additional doses of lorazepam 0.04 mg/kg (spaced 10-15 minutes apart) while awaiting a fosphenytoin loading dose. cvEEG - initiated within 1 hour of PICU arrival - revealed a symmetric but attenuated background, without epileptiform activity. She experienced short episodes of hypertension and tachycardia but was never bradycardic, nor persistently hypertensive. Her respiratory rate declined approximately 1 h after arrival to the PICU, requiring emergent intubation, at which time she had no cough or gag reflex. Post-intubation, she developed fluid-refractory hypotension necessitating inotropic and vasopressor support. She had no response to painful stimuli on central line placement and her pupils were fixed and dilated (1.5 hours into her PICU stay). She then received hypertonic saline and mannitol given concern for elevated intracranial pressure (ICP). After stabilization, she was taken emergently for a brain MRI, which revealed diffuse cerebral edema, slit-like lateral ventricles, crowding of the basilar cisterns and early downward herniation with kinking at the spinomedullary junction. There was no restricted diffusion. MRA demonstrated absent or minimal flow beyond the proximal segments of the major cerebral arteries, consistent with elevated ICP limiting cerebral perfusion. Also noted were mild, diffuse T2/FLAIR hyperintensities throughout both the deep and superficial gray matter suggesting a global, gray matter-focused process (Figure 1). She received IV dexamethasone in attempts to reduce cerebral edema. Post-MRI, she developed pulseless ventricular tachycardia requiring 2 minutes of CPR before return of spontaneous circulation. Regarding immunomodulatory therapy, she received intravenous immunoglobulin (2 g/kg), anakinra, and hydrocortisone; despite approval for remdesivir and COVID-19 convalescent plasma, these were ultimately withheld due to poor prognosis. Brain death examinations at 24 and 48 hours post-cardiac arrest were consistent with brain death. Repeat MRI between brain death examinations showed further limited cerebral perfusion and progression of uncal/tonsillar herniation. She was pronounced dead at the time of the second brain death examination.

Lumbar puncture performed immediately post-mortem yielded clear CSF with normal glucose (64 mg/dL), highly elevated protein (>1,500 mg/dL), 1 RBC, and 4 nucleated cells (50% neutrophils, 11% lymphocytes), indicating no encephalitis or meningitis. CSF culture was negative for bacteria and fungi. Extensive infectious disease testing was unrevealing for a cause of death (Table 1). Postmortem brain examination demonstrated global brain edema with mass effect leading to transtentorial herniation, rare red neurons in the cerebrum and cerebellum consistent with acute ischemic neuronal necrosis, and a very small number of chronic inflammatory cells in the leptomeninges and around a few intraparenchymal blood vessels. There was no evidence of encephalitis, large- or small-vessel vasculitis or thrombosis, or perivascular demyelination. Findings of chronic lymphocytic thyroiditis on autopsy and parotid cysts on brain MRI raised concerns for a possible underlying autoimmune condition. Thyroid testing performed on salvaged serum revealed an elevated TSH (8.924 uIU/mL; reference range 0.50-4.50) with normal free T4 (1.62 ng/dL; reference range 0.8-2.0). Thyroid antibodies were unable to be sent. Parents reported no known underlying medical/autoimmune conditions and no family history of autoimmune disease. Postmortem lung cultures grew *Candida parapsilosis*, likely related to the aspiration event. The remainder of the postmortem cultures were unremarkable (Table 1). CSF, blood, brain tissue, and bilateral lungs were negative for SARS-CoV-2 RNA by RT-PCR.

## Discussion

The pathogenetic mechanisms of the diverse neurological manifestations of COVID-19 are uncertain, though the ability of SARS-CoV-2 to invade cells using the ACE2 receptor has been confirmed.^10^ AFCE is characterized clinically by rapidly increasing ICP with neurological deterioration (GCS < 8, signs and symptoms of brainstem dysfunction) while imaging demonstrates cerebral edema and herniation. This is a rarely described but often fatal acute encephalopathic process,^8,9,11^ of which the majority of cases are associated with preceding seizures. In this case, the fulminant cerebral edema seems neither a direct viral effect, given the absence of SARS-CoV-2 RNA in the CSF and brain parenchyma, nor secondary to encephalitis, evidenced by the lack of inflammation on CSF indices and on neuropathological examination. Similarly, both cohorts of AFCE described to date note a variety of associated common pediatric infections, with a CNS pathogen identified in only a minority of cases. The heterogeneity of infections associated with AFCE suggests a gene-environment interaction in which common infections precipitate this phenotype in susceptible individuals.

Considering other potential etiologies of the patient’s diffuse cerebral edema, global hypoxic-ischemic encephalopathy seems unlikely given her continuous SpO2 monitoring and telemetry without prolonged desaturations or cardiac arrest prior to her precipitous neurological decline. Status epilepticus is also an unlikely etiology, as seizure-related cerebral edema is reported to develop over the course of 1 - 5 days rather than the 12 hours seen in this case.^12^ Arterial and venous thromboses were excluded at autopsy. Despite confirmed GAS pharyngitis, the lack of bacterial meningitis, bacteremia, or acute inflammation at postmortem examination suggests that bacterial pathogens did not directly cause her decline. The very scant chronic perivascular and leptomeningeal inflammation seen on autopsy is nonspecific, does not suggest a para-infectious cerebritis or vasculitis, and likely represents an inflammatory reaction to the brain ischemia associated with her prolonged increased intracranial pressure. Her markedly elevated CSF protein is nonspecific and confounded by the timing of lumbar puncture relative to cerebral anoxia. The chronic lymphocytic thyroiditis on autopsy and parotid cysts on MRI raise the possibility of an unrecognized autoimmune disease contributing to the severity of her case, however we were limited in the volume of scavenged blood remaining for additional etiologic testing. The pathologic systemic immune response and cytokine storm in SARS-CoV-2 infection is an increasingly recognized mediator of severe COVID-19 infection,^13–16^ and the same deranged immune response has been associated with diffuse cerebral edema, both within the context of acute viral infection^17^ and without.^18^ Finally, it has been hypothesized that perturbations of energy metabolism through mitochondrial fatty acid oxidation may lead to adenosine triphosphate utilization failure and cytotoxic brain edema.^17,19^

While much remains uncertain about the pathophysiological mechanisms of COVID-19 and AFCE, this case shows that SARS-CoV-2 childhood infection may present with AFCE in previously healthy children. This presentation is distinct from a previously reported case of fatal cerebral edema in child with SARS-CoV-2,^20^ in which elevated intracranial pressure developed in the context of SARS-CoV-2-associated multisystem inflammatory syndrome. In that case, neuropathology showed diffuse inflammation, while we did not find evidence of significant brain pathology on postmortem examination. Additionally, encephalopathy was part of our patient’s initial presentation. The previously reported case shows that fatal cerebral edema may complicate pediatric multisystem inflammatory syndrome, while our case indicates that COVID-19 infection may directly trigger AFCE. The rapid and devastating clinical course in both of these cases highlights the need for early recognition of a cerebral edema and AFCE as potential complications of COVID-19 in pediatric patients.
